# Conjugated Linoleic Acid Alleviates Hepatic Steatosis and Liver Damage in Estradiol-Induced FLHS Roosters by Reshaping Lipid Metabolism and Inhibiting the MAPK/NF-κB-Mediated Inflammation Cascade

**DOI:** 10.3390/ani16172773

**Published:** 2026-09-03

**Authors:** Xuelan Liu, Heng Zhang, Qingtao Gao, Yan Shang, Tianhong Shi, Peipei Yan, Chunyan Fu

**Affiliations:** 1 Poultry Institute, Shandong Academy of Agricultural Sciences, Jinan 250100, China; 2 Shandong Provincial Key Laboratory of Livestock and Poultry Breeding, Jinan 250100, China; 3 Jinan Key Laboratory of Poultry Germplasm Resources Innovation and Healthy Breeding, Jinan 250100, China

**Keywords:** FLHS, CLA, liver injury, lipid metabolism

## Abstract

Fatty liver hemorrhagic syndrome (FLHS) is a prevalent metabolic disease in laying hens, causing severe economic losses. A previous study showed that conjugated linoleic acids (CLA) alleviated estrogen-induced FLHS in chickens, but the mechanism remains unclear. In the present study, we found that CLA improved serum lipid homeostasis, reduced hepatic lipid accumulation, and enhanced antioxidant activity in FLHS chickens. Transcriptome analysis identified differentially expressed genes enriched in inflammatory response, lipid homeostasis, carbohydrate metabolism, and mitogen-activated protein kinase (MAPK)/peroxisome proliferator-activated receptor (PPAR)/insulin signaling pathways. Metabolome analysis detected differentially abundant metabolites enriched in bile secretion, thyroid hormone synthesis, the insulin signaling pathway, and glycerophospholipid metabolism. Integrated analyses revealed that CLA reshaped the hepatic metabolic profile by upregulating protective metabolites (such as ubiquinol) and downregulating pro-inflammatory/lipogenic metabolites (such as 15-hydroperoxyeicosa-8Z,11Z,13E-trienoate), which synergized with key gene regulation (fatty acid synthase, jun proto-oncogene, and fatty acid desaturase 2) and core pathway activity (MAPK/nuclear factor kappa-B inhibition, PPARα activation). The multi-omics study using an estradiol-induced rooster FLHS model elucidated the molecular regulatory network of CLA against hepatic steatosis and liver injury, and provided preliminary mechanistic clues for developing CLA functional additives to prevent and treat FLHS in commercial laying hens.

## Simple Summary

Fatty liver hemorrhagic syndrome (FLHS) causes substantial economic losses in laying-hen production. Numerous studies have focused on nutritional imbalance-induced FLHS and the potential underlying mechanisms. The roles of hormonal homeostasis disturbance in the onset and progression of FLHS are unclear. Conjugated linoleic acid (CLA) can relieve estradiol-induced FLHS in chickens, yet its underlying molecular mechanism remains unclear. In this study, we established an estradiol-induced FLHS model using male Hy-line white chickens and combined hepatic transcriptomics and metabolomics to explore the hepatoprotective mechanism of CLA. CLA improved serum lipid profiles, reduced hepatic lipid accumulation, and enhanced hepatic antioxidant capacity in FLHS chickens. Multi-omics analyses revealed that CLA reshaped hepatic metabolic signatures and altered gene expression related to lipid metabolism and inflammation. Key genes (FASN, JUN, FADS2) and critical metabolites (ubiquinol, L-carnitine, 15-hydroperoxyeicosa-8Z,11Z,13E-trienoate) were identified, indicating the potential involvement of MAPK/NF-κB, PPAR, and insulin signaling pathways in CLA-mediated liver protection. This work provides preliminary mechanistic clues and candidate targets for developing CLA-related functional feed additives against poultry FLHS.

## 1. Introduction

Fatty liver hemorrhagic syndrome (FLHS) represents a major non-infectious mortality factor in commercial laying hens. This metabolic disorder is manifested as excessive hepatic and abdominal fat deposition, hepatic steatosis, liver rupture, and even hepatorrhagia, resulting in substantial economic losses for the poultry industry. FLHS can be caused by nutritional, genetic, environmental, and hormonal factors [1]. It is more commonly observed in aged laying hens as a result of the intense metabolic demands at peak production [2], and can lead to significant reductions in laying rates and egg quality [3]. A Queensland survey revealed that in commercial caged hens, FLHS accounted for 40–70% of hen mortality [2]. Therefore, strategies to regulate lipid metabolism in laying hens have attracted attention because of their importance for production efficiency and health [4]. The pathogenesis of FLHS is poorly understood but appears to be highly similar to that of metabolic nonalcoholic fatty liver disease (NAFLD) in humans [5]. Numerous studies have focused on the nutritional imbalance-induced FLHS and the potential underlying mechanisms [6,7]. It is unclear about the roles of hormonal homeostasis disturbance in the onset and progression of FLHS. In order to eliminate the confounding effects originating from ovarian cyclic hormone fluctuation, egg-laying cycle and yolk-precursor metabolism, a male bird model will help us to specifically focus on estrogen-activated hepatic lipogenic, inflammatory and oxidative-stress signaling.

Conjugated linoleic acids (CLAs) are linoleic-acid derivatives carrying conjugated double bonds, drawing broad research interest owing to their capacities to modulate energy metabolism, inflammatory status and redox homeostasis across animal models [8,9]. Accumulated animal evidence indicates that CLA is deposited in multiple organs including liver, adipose depots and skeletal muscle to execute its bio-functions [10]. Two predominant isomers possess distinct bioactivities: c9,t11-CLA mainly confers anti-inflammatory properties, whereas t10,c12-CLA shows prominent anti-adipogenic potency [11]. In BV-2 microglia, both isomers suppress the production of pro-inflammatory cytokines and mediators [12]. In rats with fructose-induced NAFLD, c9,t11-CLA and t10,c12-CLA exhibit hepatoprotective activity, and the hypolipemic action of the t10,c12-CLA isomer has been observed to be more pronounced than that of c9,t11-CLA [13]. Moreover, c9,t11-CLA treatment can efficiently increase the antioxidant capacity and decrease inflammation induced by H_2_O_2_ in bovine mammary epithelial cells [14]. In poultry production, dietary CLA supplementation can modulate lipid deposition, improve antioxidant capacity and immune status, and modify egg- or meat-product fatty acid composition [15]. In our previous study, we found that CLA alleviated lipid metabolism, oxidative stress, and inflammation in estradiol benzoate-induced FLHS Hy-line male chickens [16]. However, the mechanisms have not been investigated directly.

Based on the phenotypic alleviation effect of CLA on estradiol benzoate-induced FLHS in Hy-line male chickens, the current study was conducted to investigate the hepatoprotective mechanism of CLA via hepatic transcriptome and metabolome analyses and to excavate hub genes, key metabolites, and core signaling crosstalk. The current study confirms the therapeutic potential of CLA for FLHS and provides novel insights into the effect of CLA on livestock yield and the application of functional fodder.

## 2. Materials and Methods

### 2.1. Materials

CLA, with a purity of 81% (c9, t11 CLA = 36.0%, t10, c12-CLA = 41.7%, other isomers = 3.3%), was purchased from Aohai Biologic Limited Company (Qingdao, China).

### 2.2. Ethics Statement

All of the animal experimental procedures were conducted according to the guidelines of the Institutional Animal Care and Use Committee of Shandong Academy of Agricultural Sciences (Jinan, China). The animal study was reviewed and approved by the Ethics Committees of the Laboratory Animal Center of Shandong Academy of Agricultural Sciences (SAAS-2024-G25).

### 2.3. Experimental Design and Animal Management

Male Hy-line white chickens were purchased from Shandong Haotai Experimental Animal Breeding Co., Ltd. (Jinan, China) and housed at the breeding base of the Poultry Institute of Shandong Academy of Agricultural Sciences (Jinan, China). All chickens were housed in stainless steel ladder cages (45 cm × 45 cm × 45 cm) in a controlled room on a 16L:8D illumination cycle. All of the birds were allowed free access to water and food and were subjected to an acclimatization period of one week before the formal tests, during which time they were fed a basal diet (Table 1).

The experiments were designed and conducted as previously described [16]. Briefly, one hundred and twenty 120-day-old male Hy-line white chickens of similar weight were raised in ladder cages with two birds per cage and randomly divided into two groups: a non-injected control group and a FLHS group, with 6 replicates per group and 10 birds per replicate. For FLHS model construction, roosters assigned to the FLHS group were intramuscularly administered estradiol benzoate (Ningbo Second Hormone Factory, Ningbo, China). The injection dosage was maintained at 2 mg per kg body weight, administered every other day for seven consecutive rounds [17]. As reported in our previous study [16], this estradiol-benzoate exposure successfully reproduced typical FLHS pathological features including hepatomegaly, hepatic hemorrhage and steatosis. Subsequently, 30 birds from each of the original FLHS groups, with 5 birds from each replicate, were randomly separated and received an additional diet supplemented with 0.5% CLA (CLA group, which was based on our previous studies [18]) for 8 weeks (Table 1). The remaining birds in the FLHS group stayed in the original cages and continued on the basal diet. In the current study, the FLHS group and CLA group were further observed and subjected to further analysis.

### 2.4. Sample Collection

On day 70 of the experiment, one bird from each replicate was randomly selected, with six birds per group, after fasting for 12 h, as previously described [16]. The chickens were injected intravenously with 100 mg of sodium pentobarbital per kg BW for euthanasia [15]. Blood was aseptically collected from the wing vein by using 5-mL vacuum blood tubes before the birds were euthanized by cutting the jugular vein. After the serum was naturally separated, it was centrifuged at 3000 rpm for 10 min for further separation, after which it was stored at −80 °C for subsequent testing [15]. The fresh livers were imaged and weighed. Approximately 1 cm of liver was subsequently removed and fixed in 4% paraformaldehyde for histopathological evaluation. Four small pieces of liver tissue were removed, frozen in liquid nitrogen, and stored at −80 °C for subsequent testing.

### 2.5. Serum Biochemistry Analysis

Serum biochemistry indices including triglyceride (TG), total cholesterol (TC), alanine transaminase (ALT), aspartate transaminase (AST), high-density lipoprotein cholesterol (HDL-C), and low-density lipoprotein cholesterol (LDL-C) were quantified using commercial assay kits (Nanjing Jiancheng Institute of Biological Engineering, Nanjing, China) following kit-provided operational guidelines.

### 2.6. Hepatic Biochemical and Oxidative Stress Index Analysis

The liver tissue samples were homogenized in ice-cold saline at a weight-to-volume ratio of 1 g:9 mL. Homogenates were centrifuged at 3000 rpm for 10 min, and supernatants were collected into fresh centrifuge tubes for subsequent biochemical measurement [15]. Hepatic contents of TG, TC, HDL-C, LDL-C and malondialdehyde (MDA), as well as the activities of glutathione peroxidase (GSH-Px), catalase (CAT) and superoxide dismutase (SOD), were measured using commercial assay kits from Nanjing Jiancheng Bioengineering Institute (Nanjing, China). Hepatic fat proportion was quantified through acid-hydrolysis ether-extraction following a previously published protocol [19]. Briefly, liver tissues were fully homogenized and lyophilized into powder with a vacuum freeze-dryer (Yonghe Chuangxin Electronic Technology, Qingdao, China). The lyophilized powder was loaded in a Soxhlet extractor (Hangzhou Jutong Electronics, Hangzhou, China), and lipid extraction was performed with diethyl ether in a 70–80 °C water bath over 6 h. Post-extraction residues were dried in a blast drying oven at 105 °C for 2 h, cooled inside a desiccator for 0.5 h, and weighed afterwards. Hepatic fat ratio was calculated according to the formula: (pre-extraction weight − post-extraction weight)/weight of lyophilized liver powder × 100% [15].

### 2.7. H&E and Oil Red O Staining

Liver tissue samples were rinsed with saline and immersed in paraformaldehyde solution for one week of fixation. Fixed specimens went through standard paraffin-embedding histological workflows and were sectioned at 5 µm thickness, followed by hematoxylin-eosin (H&E) staining using reagents supplied by Servicebio (Wuhan, China). Xylene washing was conducted on paraffin sections before mounting with neutral resin. For lipid visualization, frozen hepatic sections were subjected to Oil Red O ethanol staining (Servicebio, Wuhan, China). Gradient ethanol differentiation was performed, followed by hematoxylin counter-staining, and sections were sealed with neutral resin. All prepared slices were visualized and imaged under an Olympus CX-41 light microscope (Tokyo, Japan) [15].

### 2.8. Widely Targeted Metabolomics Analysis

Hepatic metabolite extraction was completed by MetWare (Wuhan, China) following their standard experimental workflows. Sample extracts were subjected to liquid chromatography–mass-spectrometry (LC-MS) detection on an UltiMate 3000 UPLC instrument (Thermo Fisher Scientific, Bremen, Germany). Mass-spectrometric acquisition was carried out on a QTRAP LC-MS/MS platform fitted with an ESI Turbo IonSpray source (Sciex, Framingham, MA, USA) under both positive- and negative-ion modes; data acquisition was managed by Analyst TF software (v1.8, Sciex, Framingham, MA, USA). Multiple reaction-monitoring (MRM) ion transitions were tracked corresponding to metabolite elution characteristics during chromatographic separation. Differentially abundant metabolites (DAMs) were screened using the criteria: variable importance in projection (VIP) ≥ 1, absolute |log_2_(fold-change)| ≥ 1, and raw *p* < 0.05. The VIP metrics were derived from orthogonal partial least-squares discriminant analysis (OPLS-DA), which was implemented in the R package MetaboAnalystR (v3.3.0). Prior to multivariate modeling, metabolite data underwent log_2_-transformation and mean-centering preprocessing. A permutation test with 200 random permutations was applied to evaluate model overfitting risk. Hypergeometric tests were adopted for KEGG pathway enrichment mapping, and pathways with false discovery rate (FDR) < 0.05 were regarded as statistically significant.

### 2.9. Transcriptomics Analysis

RNA-seq transcriptome profiling was conducted using hepatic tissue specimens. (1) Total RNA was isolated from liquid-nitrogen-frozen liver tissues with TRIzol reagent (Thermo Fisher), following the supplier’s operational protocols. RNA yield and purity were assessed on an Agilent 2100 Bioanalyzer together with the RNA 6000 Nano LabChip Kit (Agilent, Santa Clara, CA, USA). RNA samples satisfying quality specifications (260/280 ratio: 1.8–2.0; RIN > 7.0) were selected for library preparation. Each sequencing library yielded roughly 42 million clean reads, corresponding to a sequencing depth of approximately 6 Gb per sample. (2) Transcriptome libraries were built in compliance with standard Illumina RNA-seq workflows. After library quality validation, paired-end PE150 sequencing was carried out on an Illumina NovaSeq 6000 platform (LC-Bio Technology Co., Ltd., Hangzhou, China). (3) Raw sequencing reads were pre-processed to acquire clean reads. Bowtie2 (v2.3.5.1) was applied to align clean reads against the SILVA database for ribosomal RNA removal. The remaining reads were mapped to the Gallus gallus reference genome (GCF_016699485.2, NCBI) employing HISAT2 software (v2.2.1), achieving an average mapping rate of 91.4%. All samples met the criterion Q30 > 93%. Gene-level read counts were computed by FeatureCounts (v.1.6.3) based on gene coordinates from the GTF genome annotation file, generating fragments per kilobase of transcript per million mapped reads (FPKM) values for each sample [20]. (4) Principal component analysis (PCA) was implemented for linear dimensionality reduction to characterize inter-sample divergence. The edgeR package (v3.42.4) was utilized for screening differentially expressed genes (DEGs) with thresholds set as |log_2_(fold-change)| > 1.0 and *p* < 0.05. Functional enrichment analyses for Gene Ontology (GO) and Kyoto Encyclopedia of Genes and Genomes (KEGG) were completed with topGO and KOBAS (v3.0), respectively. Protein–protein interaction (PPI) networks of DEGs were constructed via the STRING database (http://string-db.org/, accessed on 10 July 2026), and network visualization was accomplished in Cytoscape 3.9.1 according to a previously published protocol [19]. Hub-gene candidates were screened with MCODE and CytoHubba plugins. For MCODE configuration: Degree Cutoff = 2 (Network Scoring); Node Score Cutoff = 0.2, K-Core = 2, Max Depth = 100 (Cluster Finding). Genes derived from module analysis were combined to constitute the final hub-gene collection.

### 2.10. RNA Extraction and Real-Time Quantitative Polymerase Chain Reaction

Total RNA was isolated from hepatic tissues collected from FLHS model roosters and CLA-intervened FLHS roosters utilizing a BioFlux total-RNA extraction kit (Hangzhou, China), in strict compliance with manufacturer-supplied protocols. RNA concentration was quantified with a K5800 micro-spectrophotometer (Kaiao Technology, Beijing, China). Purified mRNA was reverse-transcribed into complementary DNA (cDNA) employing the Evo M-MLV RT Kit with gDNA Clean for qPCR (Accurate Biotechnology Co., Ltd., Changsha, China). Primers targeting lipid-metabolism-associated candidate genes were designed via NCBI Primer-BLAST (http://www.ncbi.nlm.nih.gov/tools/primer-blast/, accessed on 8 July 2026) and chemically synthesized by Sangon Biotech (Shanghai, China). Detailed primer sequences are summarized in Table 2. Quantitative real-time PCR (qRT-PCR) reactions were carried out on a real-time PCR instrument manufactured by Hangzhou Lifereal Biotechnology (Hangzhou, China), using SYBR Green Premix Pro Taq HS qPCR Kit (Accurate Biotechnology Co., Ltd., Changsha, China). The thermal cycling profile contained an initial hot-start step at 95 °C for 30 s, then 40 amplification cycles consisting of denaturation at 95 °C for 5 s and combined annealing-extension at 60 °C for 30 s. β-actin served as the internal reference housekeeping gene. Relative mRNA abundance of target genes was calculated using the 2^−∆∆CT^ comparative quantification method.

### 2.11. Protein Extraction and Western Blotting

Total hepatic protein was harvested using mixed lysis buffer (RIPA lysis buffer: phenylmethylsulfonyl fluoride: phosphatase inhibitor = 100:1:1; Biosharp, Hefei, China). The bicinchoninic acid (BCA) assay kit (Beyotime, Shanghai, China) was adopted to determine total protein concentration. Western blot experimental workflows were implemented following a previously reported protocol [16]. In brief, identical quantities of total protein were resolved via sodium dodecyl sulfate-polyacrylamide gel electrophoresis (SDS-PAGE). Target protein levels were probed with primary antibodies against ERK1/2, p-ERK1/2, p38 MAPK, p-p38 MAPK, NF-κB, and TNFα (Cell Signaling Technology, Shanghai, China). β-Actin (Beyotime, Shanghai, China) served as the internal loading control. HRP-labeled goat anti-mouse IgG (H + L) and goat anti-rabbit IgG (H + L) secondary antibodies (Beyotime, Shanghai, China) were applied for antigen detection. Immunoreactive bands were developed with enhanced chemiluminescence substrate (Beyotime, Shanghai, China) and captured by the Clinx gel-imaging system (Clinx, Shanghai, China). The relative optical-density values of immunoblot bands were captured by Clinx (Shanghai, China) and quantified with ImageJ software (v1.54f).

### 2.12. Statistical Analysis

Statistical analysis was performed with Statistical Analysis Systems version 8.0 (SAS Institute, Cary, NC, USA). The effect of CLA on BW, tissue index, biochemical indicators, and the expression of mRNA and proteins in FLHS chickens was assessed by Student’s *t*-test. Variance homogeneity across treatment groups was assessed by Levene’s test. The normality of the distribution of the BW data was assessed by the Shapiro test. DEGs and DAMs between the two groups were identified using the R package. EdgeR applies a negative binomial model for read count data. Multiple-testing correction was performed by calculating FDR (Benjamini-Hochberg method). DEGs were defined as |log_2_(fold change)| > 1.0, ** FDR < 0.05 **, and *p* < 0.05. Multivariate statistical analysis including PCA and OPLS-DA was performed. Differentially abundant metabolites (DAMs) were screened with VIP ≥ 1 from OPLS-DA combined with univariate statistical analysis. The Benjamini-Hochberg FDR correction was applied to adjust *p*-values for multiple-comparison bias. DAMs were defined as VIP ≥ 1, |log_2_FC| ≥ 1, ** FDR < 0.05 **. The data are shown as the mean ± standard error of the mean (SEM). *p* < 0.05 was considered to indicate statistical significance. The graphics were generated using GraphPad Prism 7 (GraphPad Software, San Diego, CA, USA).

## 3. Results

### 3.1. CLA Ameliorates Hepatic Lipid Metabolism and Oxidative Stress in FLHS Chickens

**Figure 1:**
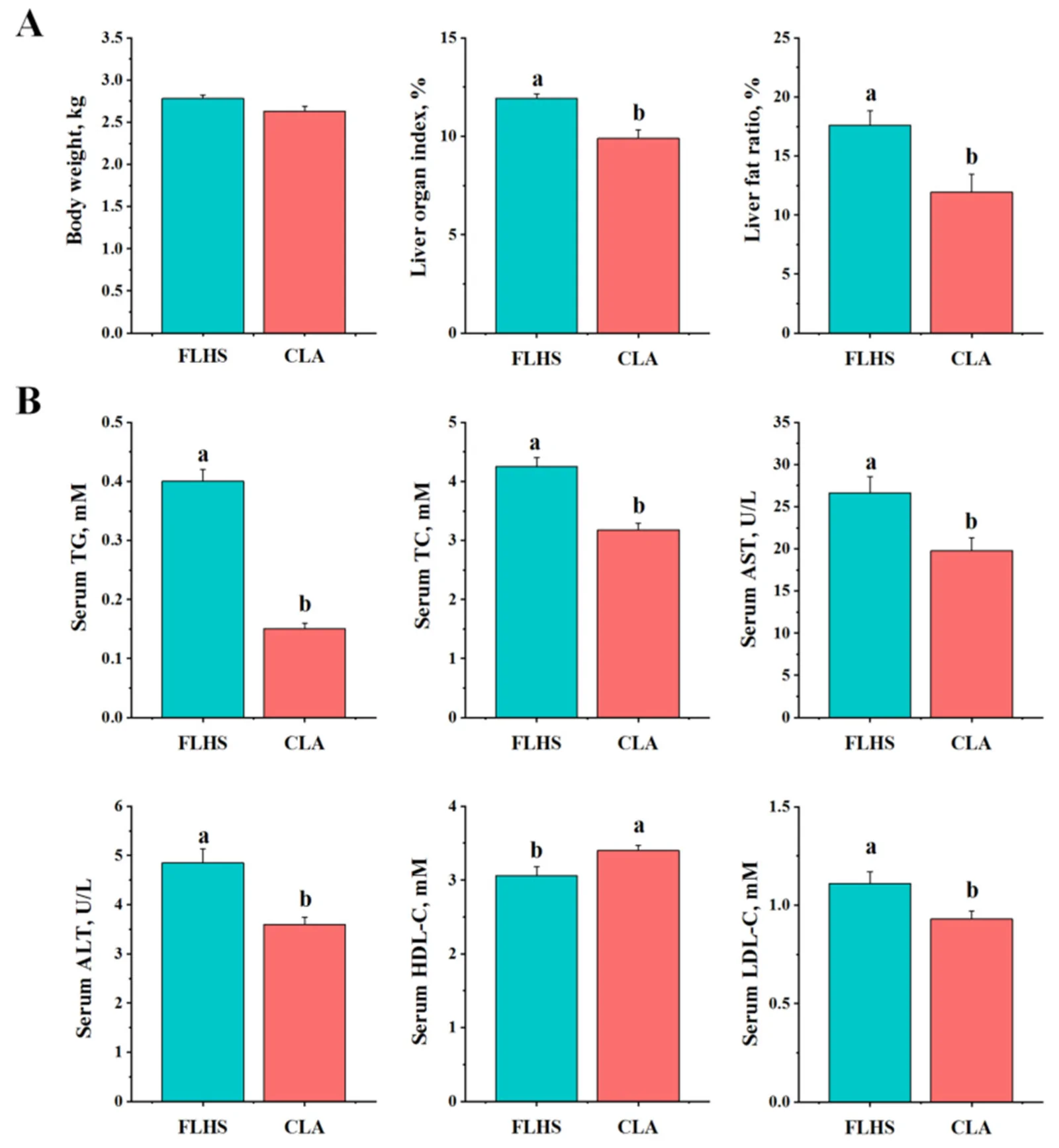
Effects of CLA on the body weight, liver organ index, liver fat ratio and serum parameters of FLHS chickens. (**A**) Effects of CLA on body weight, liver organ index, and liver fat ratio of FLHS chickens; (**B**) Effects of CLA on serum TG, TC, AST, ALT, HDL-C, and LDL-C levels of FLHS chickens. The data are displayed as means and standard deviations (means ± SEM, *n* = 6). Mean values with different letters are significantly different (*p* < 0.05). TG, triglyceride; TC, total cholesterol; AST, aspartate aminotransferase; ALT, alanine aminotransferase; HDL-C, high-density lipoprotein cholesterol; LDL-C, low-density lipoprotein cholesterol. CLA, conjugated linoleic acid.

**Figure 2:**
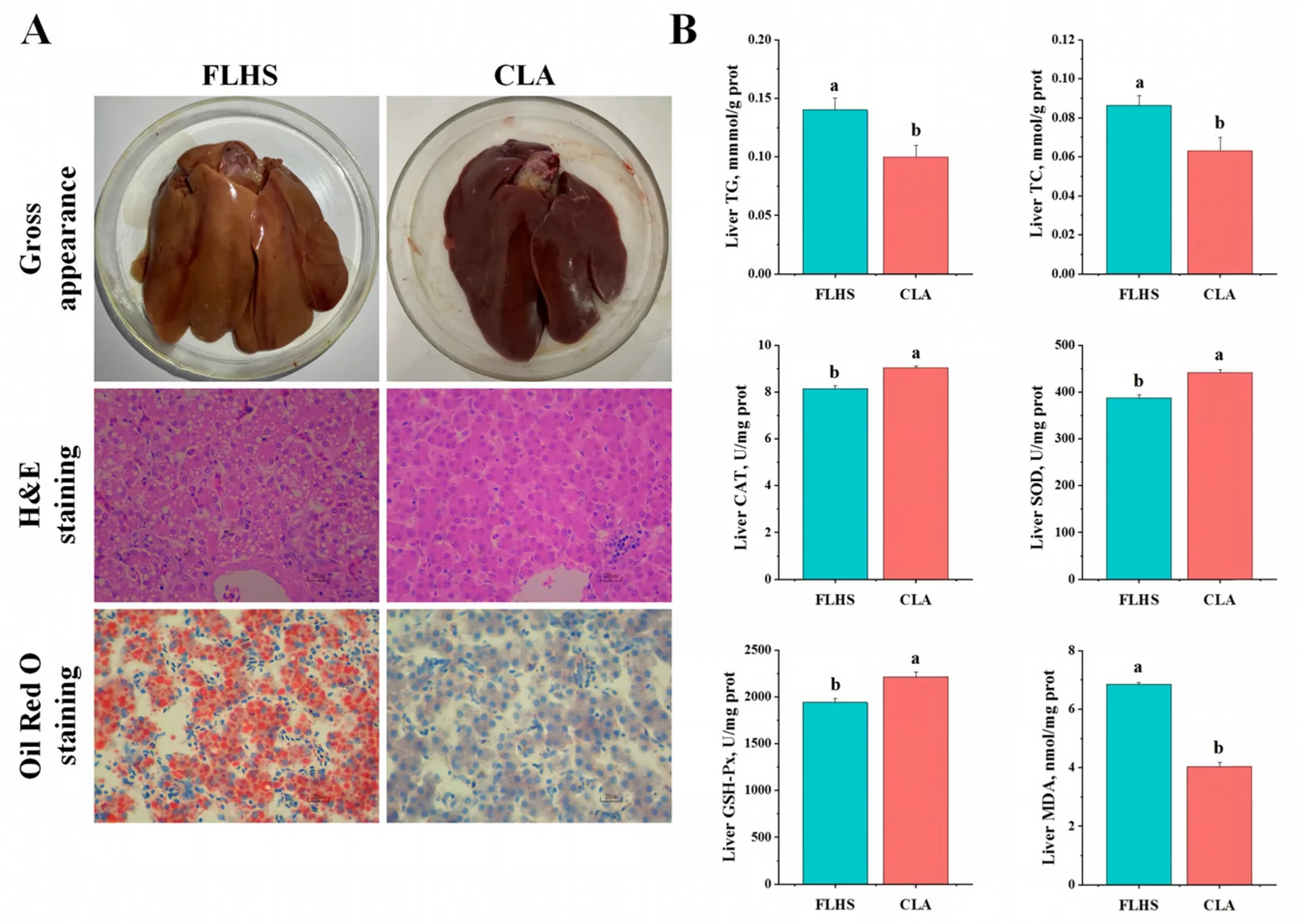
Effects of CLA on the morphology, lipid parameters, and antioxidant parameters of the liver in FLHS chickens. (**A**) Gross appearance, H&E staining (magnification: 400×), and Oil Red O staining (magnification: 400×) of livers. (**B**) Effects of CLA on levels of hepatic TG, TC, and MDA, and activity of CAT, SOD, and GSH-Px of FLHS chickens. The data are displayed as means and standard deviations (means ± SEM, *n* = 6). Mean values with different letters are significantly different (*p* < 0.05). TG, triglyceride; TC, total cholesterol; MDA, malondialdehyde; CAT, catalase; SOD, superoxide dismutase; GSH-Px, glutathione peroxidase.

To evaluate the therapeutic effects of CLA on FLHS, we treated FLHS chickens with a 0.5% CLA mixture for a period of 8 weeks. The results showed that body weight (BW) was similar in the FLHS group and CLA-treated group (*p* = 0.0973); however, the liver organ index and liver fat ratio were significantly decreased in the CLA group (Figure 1A, *p* = 0.0155, 0.0018). Furthermore, CLA treatment decreased serum TG, TC, AST, ALT, and LDL-C levels and increased serum HDL-C levels (Figure 1B, *p* < 0.05). Additionally, the physical gross appearance of the livers was markedly different between the FLHS and CLA groups (Figure 2A). Livers in the FLHS group exhibited obvious fatty liver characteristics, including large size, friability, softness, and a color varying from tan to yellow, consistent with previous reports [16]. Moreover, CLA treatment induced the liver to exhibit a typical reddish-brown color of a normal liver. In addition, histological examination of H&E-stained liver sections revealed many lipid droplets in hepatocytes, as well as fatty lesions, severe hepatic vacuolization, a disorganized hepatocyte structure, and inflammatory cell infiltration, in the FLHS group compared with the CLA group. Furthermore, compared with the FLHS group, the CLA group exhibited decreased liver TG, TC, and MDA levels and increased activity of antioxidant enzymes such as CAT, SOD, and GSH-Px in the liver (Figure 2B).

### 3.2. Identification and Functional Enrichment of Differentially Expressed Genes

**Figure 3:**
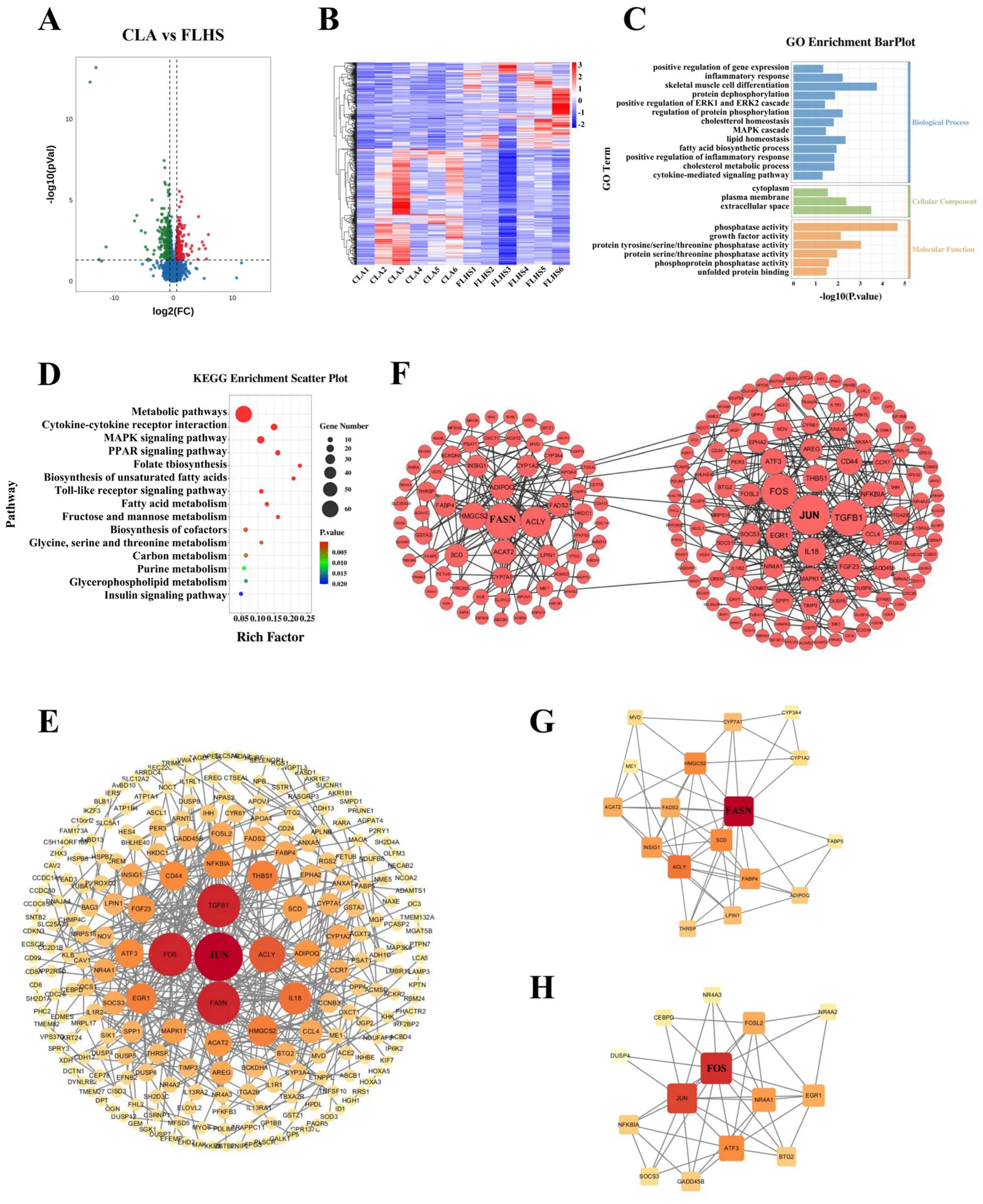
Differentially expressed mRNAs in the liver. (**A**) Volcano plot of differentially expressed genes (DEGs). Green represents downregulated genes, and red represents upregulated genes. (**B**) Hierarchical cluster analysis of DEGs. Blue represents downregulated genes, and red represents upregulated genes. (**C**) GO enrichment analysis of the DEGs. The top 15 significantly enriched terms in the biological process, molecular function, and cellular component categories are listed. When the number of significantly enriched terms was <15, all terms are shown. (**D**) KEGG pathway functional enrichment analysis of the DEGs. The top 15 significantly enriched pathways are listed. (**E**,**F**) Protein–protein interaction network of DEGs in the liver between the CLA and FLHS groups. (**G**,**H**) According to the MCODE plugin analysis, two significant gene clusters were identified. M1–M6: the samples in the FLHS group.

A total of 563 differentially expressed genes (DEGs) were detected, of which 253 and 310 were downregulated and upregulated, respectively (Figure 3A). Hierarchical clustering analysis revealed distinct mRNA expression profiles between liver samples from the two groups (Figure 3B), which confirmed that CLA exerts a regulatory effect on the liver of FLHS chickens. To clarify the biological characteristics of these DEGs, GO and KEGG pathway analyses were conducted. The most enriched terms in the biological process (BP), molecular function (MF), and cellular component (CC) categories are shown in Figure 3C. The DEGs were enriched in various BPs, including inflammatory response, cholesterol homeostasis, and lipid homeostasis, which were highly consistent with the nutritional physiology of FLHS. In addition, KEGG pathway analyses revealed that the DEGs were mainly involved in metabolic pathways, cytokine–cytokine receptor interactions, the MAPK signaling pathway, the PPAR signaling pathway, and fatty acid metabolism (Figure 3D). The majority of the above-mentioned KEGG pathways were associated with lipid and carbohydrate metabolism, thus highlighting the core role of CLA in rebalancing disrupted energy metabolism in FLHS chickens, laying a molecular foundation for CLA against FLHS. The upregulated and downregulated genes enriched in the above-mentioned nine pathways are listed in Table 3. Notably, CLA exerted significant regulation of fatty acid metabolism by downregulating key de novo fatty acid synthesis genes (FASN, stearoyl-CoA desaturase (SCD), and malic enzyme 1 (ME1)), and upregulating fatty acid oxidation and desaturation genes FADS2 and FADS1, thereby alleviating hepatic lipid accumulation. In addition, CLA modulated the inflammation response by inducing anti-inflammatory cytokine receptor genes, such as transforming growth factor beta 1 (TGFB1), C-C motif chemokine ligand 21 (CCL21), and interleukin 13 receptor subunit alpha 2 (IL13RA2), and reducing the expression of pro-inflammatory factor genes and inflammatory receptor genes, including IL1R1, IL1R2, IL18 and NF-κB inhibitor alpha (NFKBIA), and therefore mitigated liver inflammatory stress.

To investigate the interactions between the proteins encoded by the DEGs in the CLA group, a PPI network was constructed using the STRING database and presented via Cytoscape, with two large networks and two hub genes (FASN and JUN) being revealed (Figure 3E,F). According to the MCODE plugin analysis, two significant gene clusters were identified (Figure 3G,H). These clusters were defined as Cluster 1, which contained 17 proteins (including FASN, SCD, and FADS2) (Figure 3G), and Cluster 2, which consisted of 14 genes (including JUN, FOS, and NFKBIA) (Figure 3H). Two major gene clusters containing hub genes FASN and JUN were identified from the PPI network and MCODE module analysis.

**Figure 4:**
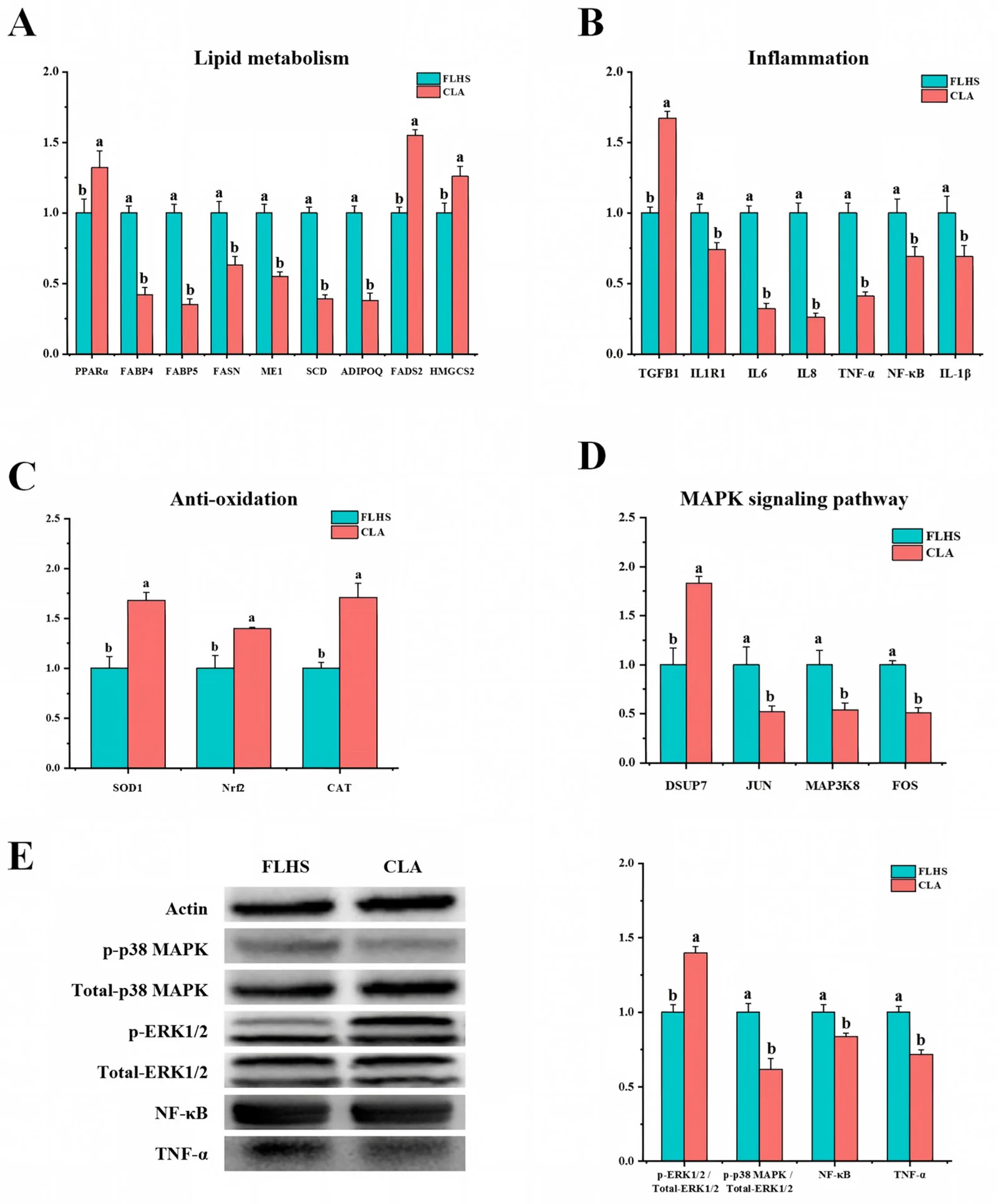
Effects of CLA on the relative expression of genes and proteins related to lipid metabolism, inflammatory regulation, antioxidant defense, and MAPK signaling pathway regulation. The values are represented as the mean ± SEM (*n* = 6). Mean values with different letters are significantly different (*p* < 0.05). (**A**–**D**), the relative mRNA expression of genes related to lipid metabolism, inflammatory regulation, antioxidant defense, and MAPK signaling pathway regulation; (**E**) the relative protein expression of p-ERK1/2, p-p38 MAPK, NF-κB, and TNF-α. ADIPOQ, adiponectin; CAT, catalase; DUSP7, dual specificity phosphatase 7; ERK, extracellular signal-regulated kinase; FABP4/5, fatty acid binding protein 4/5; FADS2, fatty acid desaturase 2; FASN, fatty acid synthase; FOS, FBJ osteosarcoma oncogene; HMGCS2, 3-hydroxy-3-methylglutaryl-CoA synthase 2; IL-1β, interleukin-1 beta; IL1R1, interleukin 1 receptor type 1; IL6/8, interleukin 6/8; JUN, Jun proto-oncogene; MAP3K8, mitogen-activated protein kinase kinase kinase 8; ME1, malic enzyme 1; NF-κB, nuclear factor kappa-B; Nrf2, nuclear factor erythroid 2-related factor 2; PPARα, peroxisome proliferator-activated receptor α; SCD, stearoyl-CoA desaturase; SOD1, superoxide dismutase; TNF-α, tumor necrosis factor alpha; TGFB1, transforming growth factor beta 1.

To validate the reliability of the above findings and further confirm the expression patterns of key regulatory genes, qRT-PCR was performed on the same set of liver samples used for transcriptome sequencing. Based on the principles of functional relevance to FLHS pathogenesis and significance of expression differences in transcriptome data, 23 candidate genes were selected, covering PPAR signaling pathway regulation and lipid metabolism (PPARα, FABP4, FABP5, FASN, ME1, SCD, ADIPOQ, FADS2, and HMGCS2), inflammatory regulation (TGFB1, IL1R1, IL6, IL8, TNFα, NF-κB, and IL-1β), antioxidant defense (SOD1, Nrf2, and CAT), and MAPK signaling pathway regulation (DUSP7, JUN, FOS, and MAP3K8). As shown in Figure 4, the qPCR results demonstrated a high degree of concordance with the transcriptome sequencing data in terms of gene expression trends. Compared to the FLHS group, CLA treatment significantly decreased the mRNA levels of lipid synthesis-related genes (Figure 4A, FABP4, FABP5, FASN, ME1, SCD, and ADIPOQ), and pro-inflammatory genes (Figure 4B, IL1R1, IL6, IL8, TNFα, NF-κB, and IL-1β) (*p* < 0.05), while lipid catabolism genes (FADS2 and HMGCS2), anti-inflammatory genes (TGFB1), and antioxidant genes (Figure 4C, SOD1, Nrf2, and CAT) were remarkably upregulated (*p* < 0.05). In order to further investigate the regulatory roles of the PPAR signaling pathway and the MAPK signaling pathway, we selected several key target genes in the above two pathways and detected their mRNA expression levels. We found that CLA increased the mRNA expression of PPARα and DUSP7, but decreased the mRNA expression of JUN, MAP3K8, and FOS compared to the FLHS group (Figure 4D, *p* < 0.05), which was consistent with the transcriptome sequencing data. To confirm the regulatory role of CLA on the MAPK signaling pathway and the inflammatory response in FLHS chickens in alleviating liver injury, we further detected the protein expression of p-ERK1/2, p-p38 MAPK, NF-κB, and TNFα. The results showed that CLA increased the expression of p-ERK1/2/total-ERK1/2, but decreased the expression of p-p38 MAPK/total-p38 MAPK, NF-κB, and TNFα in FLHS chickens (Figure 4E, *p* < 0.05). Consistent expression trends were observed between qRT-PCR/Western-blot data and transcriptome profiling.

### 3.3. Analysis of Differentially Abundant Metabolites

**Figure 5:**
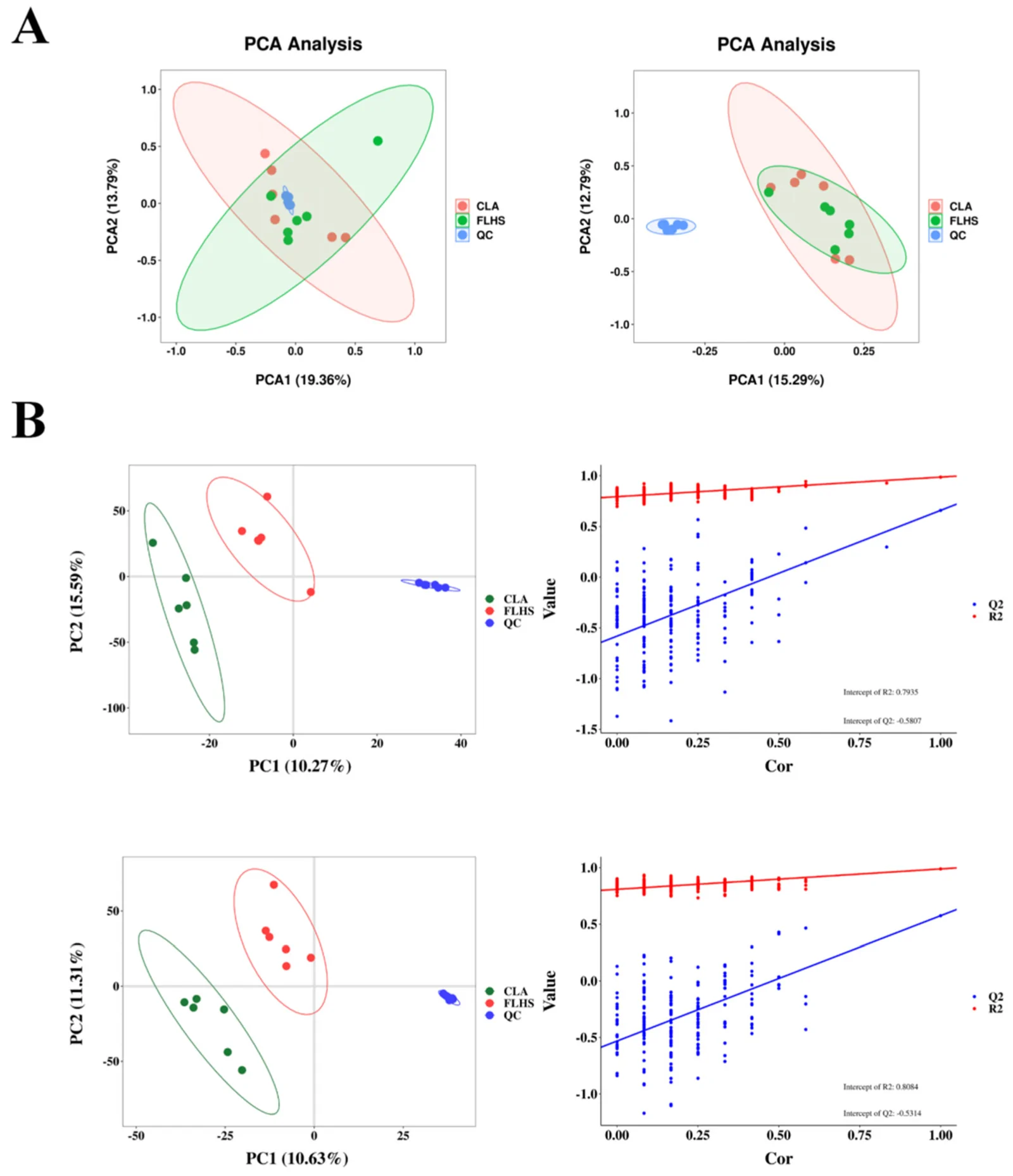
PCA and PLS-DA results of two groups of samples and QC samples (positive and negative modes). (**A**) PCA results of two groups of samples and QC samples in the positive (**left**) and negative (**right**) ion modes; (**B**) PLS-DA model score diagram (**left**) and model validation diagram (**right**) for the two groups of samples in the positive ion mode (**up**) and negative ion mode (**down**). PCA, principal component analysis; PLS-DA, partial least squares discriminant analysis.

A total of 28,395 peaks were identified, with 3077 metabolites being annotated. PCA showed concentrated QC samples in both positive and negative ion modes. It showed partial overlap between experimental groups, while tightly clustered QC samples verified the stability of the instrument detection system; the variance contribution ratios of PC1 and PC2 were explained in detail below (Figure 5A). PLS-DA revealed good repeatability of samples in the two groups (Figure 5B), and model validation (permutation test, *n* = 200) with R^2^ intercepts of 0.7935 (positive) and 0.8084 (negative) and Q^2^ intercepts of −0.5807 (positive) and −0.5314 (negative) indicated no overfitting, ensuring the data’s suitability for subsequent validation.

**Figure 6:**
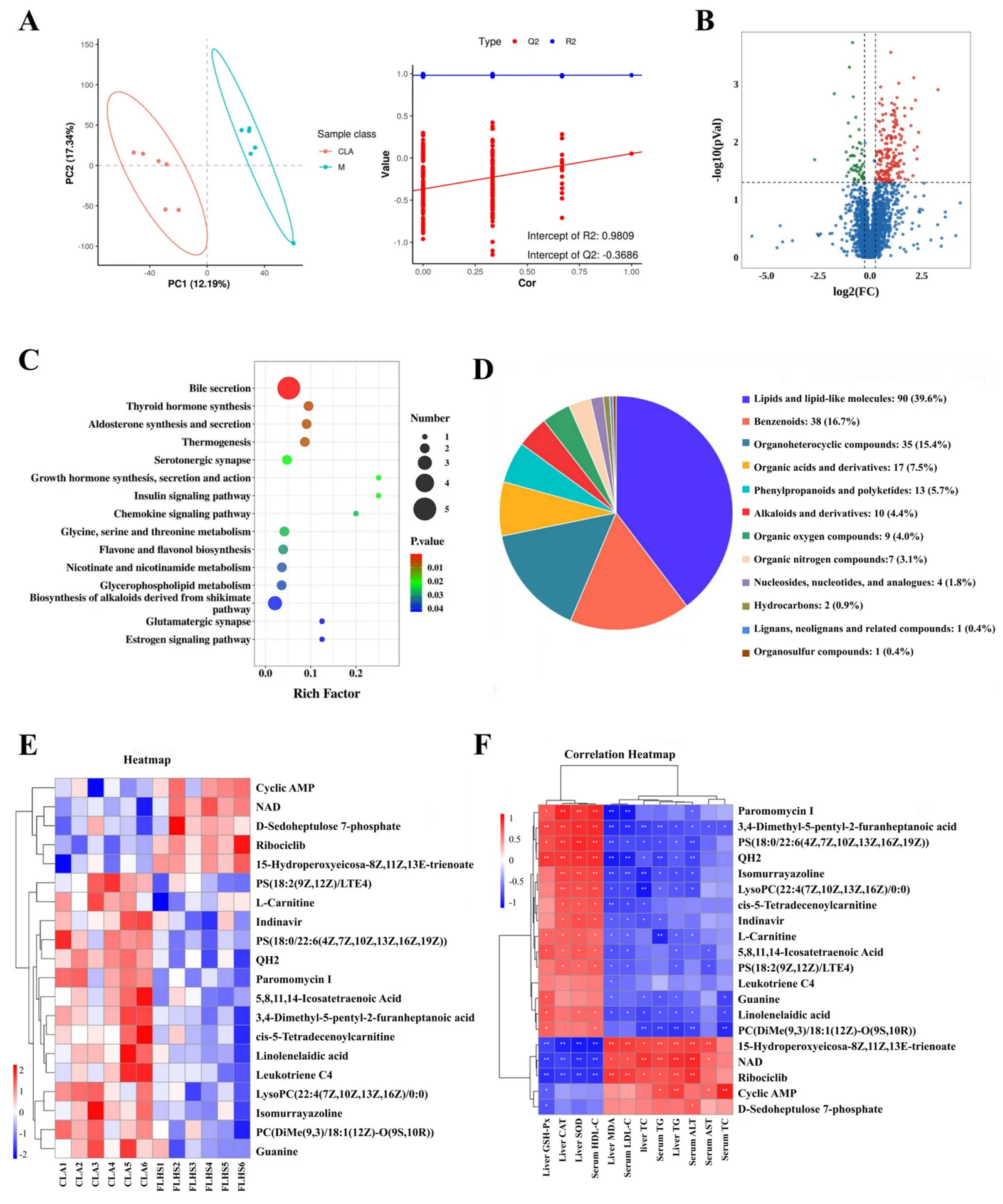
Metabolome analysis of the livers in the CLA and FLHS groups. (**A**) PLS-DA model score diagram for the differentially abundant metabolites (DAMs). (**B**) Analysis of DAMs between the CLA and FLHS groups. Red indicates an increase in metabolite content, blue indicates a decrease in metabolite content, and blue indicates no significant difference. (**C**) KEGG pathway functional enrichment analysis of the DAMs. (**D**) Classification of DAMs in the HMDB database. (**E**) Correlation analysis between DAMs and samples. (**F**) Correlation analysis between DAMs and disease indices. “*” and “**” represent *p* < 0.05 and *p* < 0.01, respectively, which indicate significant correlations between DAMs and disease indices. TG, triglyceride; TC, total cholesterol; HDL-C, high-density lipoprotein cholesterol; LDL-C, low-density lipoprotein cholesterol; AST, aspartate aminotransferase; ALT, alanine aminotransferase; CAT, catalase; SOD, superoxide dismutase; MDA, malondialdehyde; GSH-Px, glutathione peroxidase.

Based on VIP > 1 and *p* < 0.05, 163 differentially abundant metabolites (DAMs) were identified in the CLA group compared with the FLHS group (133 upregulated and 30 downregulated) (Figure 6A,B). KEGG enrichment showed that DAMs were mainly enriched in bile secretion; thyroid hormone synthesis; aldosterone synthesis and secretion; growth hormone synthesis, secretion, and action; insulin signaling; chemokine signaling; and glycerophospholipid metabolism (Figure 6C), which were closely linked to lipid homeostasis and inflammatory regulation, and suggested the core intervention targets of CLA on FLHS-induced hepatic metabolic disturbance.

HMDB annotation indicated lipids and lipid-like molecules accounted for 39.6% of DAMs, benzenoids accounted for 16.7%, organoheterocyclic compounds accounted for 15.4%, organic acids and derivatives accounted for 7.5%, phenylpropanoids and polyketides accounted for 5.7%, alkaloids and derivatives accounted for 4.4%, organic oxygen compounds accounted for 4.0%, and the remaining compounds accounted for 6.6% (Figure 6D), among which lipids and lipid-like molecules was the largest category, consistent with FLHS’s pathological feature of lipid metabolism disorder. Heatmap analyses of the DAMs were used to visually observe the different expression patterns of the compounds. The expression levels of the DAMs in the CLA group significantly differed from those in the FLHS group (Figure 6E). Notably, the antioxidant properties correlated positively with lipid metabolism-related metabolites, such as paromomycin I, 3,4-dimethyl-5-pentyl-2-furanheptanoic acid, PS (18:0/22:6) and QH2 (Figure 6F), while lipid metabolism was positively correlated with glycerophospholipid- and bile secretion-related metabolites, such as 15-hydroperoxyeicosa-8Z,11Z,A3E-trienoate, NAD, ribociclib, and cyclic AMP, and negatively correlated with QH2, isomurrayazoline, lyso PC (18:2), L-carnitine, guanine, and PC (di-me (9, 3)/18:1). Glycerophospholipids are key components of cell membranes, and their metabolism disorder exacerbates hepatic lipid accumulation. Bile secretion promotes lipid emulsification and excretion. These results suggested that CLA alleviated FLHS-induced liver injury via improving lipid transport and excretion. Additionally, the correlation analysis revealed associations between metabolite abundance and hepatic phenotypic indicators.

### 3.4. Integrated Transcriptome and Metabolome Analysis

**Figure 7:**
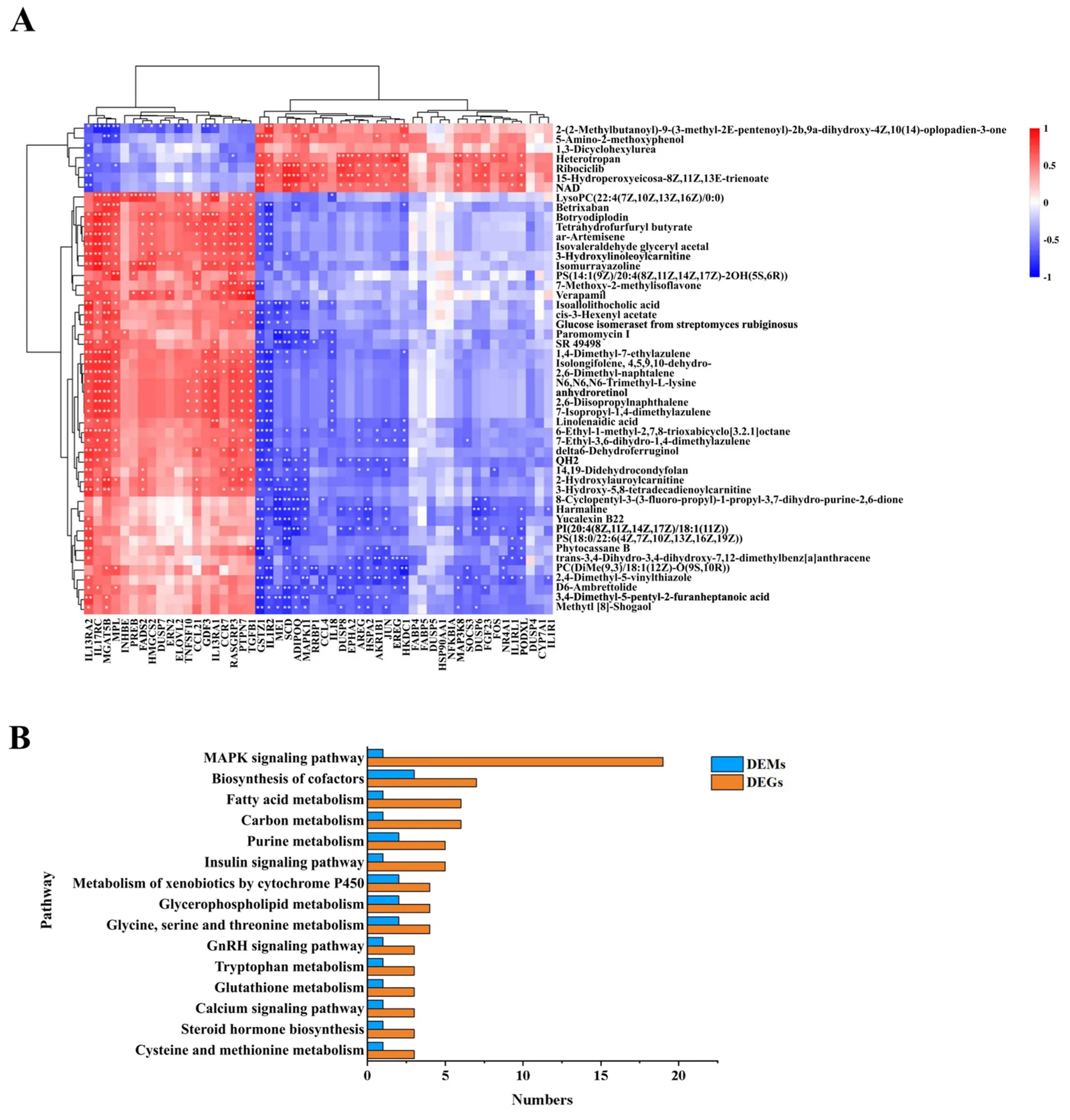
Combined transcriptome and metabolome analysis. (**A**) Heatmap of correlations between differentially expressed genes (DEGs) and differentially abundant metabolites (DAMs). Red and blue represent positive and negative correlations between transcriptomics and metabolomics, respectively. “*” and “**” represent *p* < 0.05 and *p* < 0.01, respectively, which indicate significant differences between genes and metabolites. (**B**) The transcriptome and metabolome co-enrichment of the KEGG pathway.

To further analyze the potential regulatory mechanism through which CLA alleviates liver injury in FLHS hens, Pearson correlation analysis was performed on the DEGs and DAMs. As shown in Figure 7A, the correlation between the top 50 DEGs and DAMs further highlights the obtained results. In particular, the association of each gene with each metabolite was determined. To further analyze the effects of CLA on FLHS chickens, a comprehensive analysis of metabolomic and transcriptomic data was performed. DEGs and DAMs were annotated into 15 pathways (Figure 7B). Among these pathways, the MAPK signaling pathway, fatty acid metabolism, carbon metabolism, purine metabolism, the insulin signaling pathway, and glycerophospholipid metabolism were annotated (Figure 7B).

## 4. Discussion

FLHS can spontaneously develop as fatty liver and hepatic steatosis in animals on a high-energy and high-protein diet [1,21]. CLA has received considerable attention because of its potential to regulate lipid metabolism and oxidative resistance in animals [18,22,23]. In the current study, we observed that CLA intervention decreased lipid accumulation, eliminated liver injury, and increased antioxidant activity in FLHS chickens. It was confirmed that CLA acted in its regulatory role through a multi-level network, which includes metabolic reprogramming and the regulation of gene expression and signal transduction in the liver of FLHS chickens. The cross-talk between ERK/PPARα and MAPK/NF-κB pathways is the key mechanism for CLA to alleviate FLHS. The association of specific genes with specific metabolites provided a new target for the investigation of the therapeutic regimen of FLHS.

### 4.1. CLA Ameliorated Lipid Metabolism and Oxidative Stress, and Therefore Alleviated Liver Injury in FLHS Chickens

The liver is the primary organ responsible for lipid metabolism in poultry and plays a crucial role in various processes related to lipid metabolism, such as fatty acid absorption, de novo synthesis, and oxidative decomposition of fatty acids. As reported in NAFLD models, insulin resistance, oxidative stress, and lipid peroxidation aggravate liver cell damage by altering the expression of genes involved in these biological processes [24]. In our current study, lipid metabolism disorder in the FLHS model was effectively reversed by CLA treatment through decreases in serum TG, TC, and LDL-C levels, as well as decreases in hepatic TC and TG levels, consistent with the findings of previous studies [18,22,23]. Second, CLA treatment enhanced hepatic anti-oxidase activity by increasing the activities of CAT, SOD, and GSH-Px and decreasing the level of MDA in the liver of FLHS chickens, suggesting that CLA treatment decreased oxidative stress in FLHS chickens. A previous study revealed that t10,c12-CLA could induce the activities of the antioxidant enzymes SOD and CAT, enhance free radical scavenging in hepatocytes, and reduce intracellular ROS production without increasing MDA overproduction [25], which was consistent with the results of the current study. Third, CLA treatment alleviated hepatic vacuolization, disorganized hepatocyte structure, and inflammatory cell infiltration in FLHS chickens, reflecting a reduction in liver damage. These results suggest that CLA plays a regulatory role in lipid metabolism and oxidative stress, along with further alleviating liver injury in FLHS chickens.

### 4.2. The MAPK and PPAR Signaling Pathways Participated in CLA’s Hepatoprotective Effects in FLHS Chickens

Our work further elucidates the mechanism through which CLA alleviates liver injury in chickens with FLHS. Several studies have demonstrated that metabolic pathway disturbances (including the desaturation and elongation of fatty acids, disorders of glucose and amino acid metabolism, and oxidative phosphorylation) may cause inflammation and contribute to the pathogenesis of FLHS [26,27,28]. To investigate the molecular mechanisms through which CLA relieved liver injury in FLHS chickens, we performed comparative transcriptomic analyses. We observed that the DEGs were enriched in fatty acid and glucose metabolism, confirming the regulatory role of CLA in glucolipid metabolism in chickens with FLHS. KEGG enrichment analysis revealed that the DEGs were significantly enriched in the MAPK signaling pathway, PPAR signaling pathway, and insulin signaling pathway, which are closely linked to FLHS development. Additionally, PPI network analysis identified FASN and JUN as the hub genes, with two functional modules that collectively mediate CLA’s protective effects through metabolic regulation and inflammatory suppression.

In MCODE 1, genes involved in fatty acid synthesis, β-oxidation, and lipid transport, including FASN, SCD, FADS2, HMGCS2, and FABP4, were enriched, which confirmed their central role in CLA-regulated lipid metabolic homeostasis in FLHS chickens. As a core regulator of lipid metabolism, FASN catalyzes the conversion of dietary carbohydrates into fatty acids [29], while SCD, as a key marker of lipid metabolism, accelerates fatty liver progression by promoting lipid deposition [30,31]. HMGCS2 is the key rate-limiting enzyme of ketogenesis and catalyzes the breakdown of β-oxidation-derived acetyl-CoA and acetoacetyl-CoA to produce β-hydroxy β-methylglutaryl-CoA and free coenzyme A [32]. These findings indicated that CLA might modulate the catalytic activity of key enzymes in fatty acid metabolism, thereby reducing abnormal lipid accumulation in the liver of FLHS chickens. Notably, the PPAR signaling pathway, wherein FASN acts as a downstream target, has been shown to play a critical role in FLHS development, as its disruption exacerbates hepatic steatosis [24]. These results suggested that CLA alleviated glucolipid metabolism disorders in FLHS birds, which might be regulated by the FASN-mediated PPAR signaling pathway.

In MCODE 2, genes involved in the MAPK signaling pathway, toll-like signaling pathway, and insulin signaling pathway were enriched, such as JUN, FOS, NFKBIA, SOCS3, NR4A1, and DUSP7, which highlighted the regulatory role of CLA in inflammatory and insulin metabolism. JUN (c-Jun), a component of the AP-1 transcription factor complex (alongside FOS), promotes hepatic steatosis by upregulating lipogenic gene expression [33]. NFKBIA encodes IKBα, which inhibits NF-κB (a master regulator of inflammation) by sequestering it in the cytoplasm and blocking its transcriptional activity [28,34]. Meanwhile, SOCS3, a key transcription factor in lipid accumulation regulation, induces hepatic insulin resistance and fatty acid synthesis when overexpressed [35,36]. Given that MAPK pathway activation exacerbates hepatic steatosis and mitochondrial dysfunction, key features of metabolic-associated steatotic liver disease (MASLD) progression [37,38], our results suggested that CLA mitigated inflammation and insulin resistance in FLHS birds, which might be mediated by the JUN-mediated MAPK signaling pathway.

Classical MAPK pathways, such as p38 MAPK and ERK, interact closely with NF-κB to regulate inflammatory processes [39]. In our study, CLA treatment reduced p38 MAPK phosphorylation, NF-κB activity, and the expression of downstream pro-inflammatory genes in FLHS chickens. Previous findings showed that blunting p38 MAPK activity alleviates liver fibrosis by suppressing NF-κB and reducing lipid accumulation [40,41]. Additionally, ERK phosphorylation exerted dual protective effects, as it inhibited NF-κB to promote nerve repair [42] and downregulated FASN expression to reduce lipid synthesis [43]. In primary chicken hepatocytes, t10,c12-CLA has been reported to activate the ERK1/2 signaling pathway to suppress excessive lipogenic gene expression [44]. Notably, ERK signaling also cross-talks with the PPARα pathway, an important regulator of fatty acid β-oxidation, inflammation, and liver carcinogenesis [45]. Our data showed that CLA increased ERK1/2 phosphorylation and PPARα mRNA expression, accompanied by reduced lipid synthesis, inflammation, and oxidative stress, which indicated that the hepatoprotective effects of CLA might be enhanced by the interaction of the PPARα pathway and MAPK/NF-κB pathways.

### 4.3. Metabolomics Revealed the Key Metabolites Participating in CLA’s Hepatoprotective Effects in FLHS Chickens

Metabolomics enables systematic analysis and identification of small-molecule metabolites (<1.5 kDa) such as glycolipids, short peptides, and nucleotides in cells and tissues, serving as a cornerstone of modern biological research. In the current study, 163 DAMs were identified in the liver of FLHS chickens treated with CLA, including 133 upregulated and 30 downregulated. KEGG enrichment analysis highlighted key pathways, such as bile secretion, thyroid hormone synthesis, the insulin signaling pathway, and glycerophospholipid metabolism, which were also enriched in transcriptomic analysis of DEGs.

Ubiquinol (QH_2_) is an essential, lipid-soluble, redox component of the mitochondrial respiratory chain and exerts antioxidant effects in a number of membrane and biological systems by preventing peroxidative damage to lipids [46,47]. In the current study, CLA increased QH_2_ levels in the liver of FLHS chickens, and correlation analysis showed that QH_2_ had a negative correlation with the expression of SCD, ADIPOQ, DUSP8, and JUN. A previous study showed that coenzyme Q (the oxidized form of QH_2_) suppressed tissue dysfunction by inhibiting MAPK signaling pathway activation and enhancing PPARα-mediated fatty acid β-oxidation [48]. In the current study, CLA inhibited the phosphorylation of p38 MAPK and upregulated the expression of PPARα. These findings raised the possibility that CLA may enhance hepatic QH2 abundance to counteract oxidative stress. Based on previous reports, accumulated QH2 could potentially suppress excessive p38 MAPK activation and cooperate with PPARα signaling to restore hepatic lipid homeostasis, which remains to be experimentally validated.

L-carnitine, a vitamin-like substrate, facilitates the transport of fatty acids into mitochondria for β-oxidation and has been shown to alleviate liver injury in high-fat diet-induced NAFLD mice by regulating lipid metabolism, antioxidant capacity, and inflammation [49,50]. In the current study, CLA increased the L-carnitine level in the liver of FLHS chickens, which was consistent with previous reports. Combined with the expression of fatty acid β-oxidation-related genes, these results indicated that CLA enhanced fatty acid β-oxidation by increasing L-carnitine synthesis, which might further reduce lipid accumulation in the liver of FLHS chickens.

Linolenelaidic acid is an omega-fatty acid that can ameliorate liver fibrosis by inhibiting TGFβ signaling in hepatic stellate cells and decreasing hepatic lipid accumulation [51]. PS (18:0/22:6 (4Z, 7Z, 10Z, 13Z, 16Z, 19Z)), also known as phosphatidylserine, is required for the proper transbilayer distribution of cholesterol [52]. In the current study, CLA increased linolenelaidic acid and PS (18:0/22:6 (4Z, 7Z, 10Z, 13Z, 16Z, 19Z)) levels in the liver of FLHS chickens, which suggested their role in improving lipid structure and reducing liver damage. LysoPC (22:4 (7Z, 10Z, 13Z, 16Z)/0:0) is a kind of lysophosphatidylcholine, which contains a docosatetraenoic acid (DTA), the precursor of docosatetraenoyl ethanolamine (DEA). A previous study found that DTA exhibited protective effects against persistent gestational hyperthyroidism and inflammation throughout pregnancy [53]. In the current study, CLA increased lysoPC (22:4 (7Z, 10Z, 13Z, 16Z)/0:0) levels in the liver of FLHS chickens, which was positively correlated with FADS2. In the liver, FADS2 promotes the synthesis of arachidonic acid from linoleic acid, which is further converted to DTA by elongase. The correlation suggested that CLA might regulate FADS2 transcription to enhance lysoPC (22:4 (7Z, 10Z, 13Z, 16Z)/0:0) and subsequent DTA synthesis, which might further exert anti-inflammatory and hepatoprotective effects. Further verification was needed.

15-Hydroperoxyeicosa-8Z,11Z,13E-trienoate (15s-HPETE) is a lipid peroxide and the oxidative precursor of 15-hydroxyeicosatetraenoic acid (15-HETE) [54]. Emerging evidence has suggested that 15s-HPETE can induce membrane lipid peroxidation and free radical generation, which might further exert proinflammatory properties and contribute to endothelial cell injury [55,56]. In the current study, CLA decreased 15s-HPETE levels in the liver of FLHS chickens. PPARs are members of the nuclear receptor superfamily, act as ligand-inducible transcription factors, and play crucial roles in glucose and lipid metabolism [57,58]. It was reported that 15-HETE can induce PPARs coactivator binding and transcriptional activation in vivo, and further activate the downstream target gene expression, such as ADIPOQ, SCD, and ME1 [59]. In the current study, we found that the expression of 15s-HPETE was positively correlated with the expression of ADIPOQ, SCD, and ME1, which suggested that CLA might downregulate the expression of 15s-HPETE and further inhibit the PPARs-mediated glucolipid metabolism in FLHS chickens.

### 4.4. Integration Analysis Revealed the Potential Crosstalk of Signaling Pathways and Regulatory Nodes of CLA-Alleviated Liver Injury in FLHS Chickens

Integrated transcriptome–metabolome approaches have been increasingly applied to decode complex hepatic metabolic perturbations in poultry, providing a powerful strategy to uncover key genes and metabolite signatures underlying avian fatty-liver pathologies [60,61]. Transcriptomic and metabolomic data were co-enriched in the insulin signaling pathway, which may suggest potential pathway-level associations for CLA intervention rather than proven regulatory effects, highlighting possible links between CLA and glucose metabolism. Metabolomic analysis showed that CLA downregulated D-sedoheptulose 7-phosphate, a critical intermediate bridging the pentose phosphate pathway and glycolysis that links energy metabolism to immune regulation [62,63]. The reduction of this intermediate directly affects the flux of glucose metabolism, reducing excessive energy accumulation in the liver. Transcriptomic analysis identified SOCS3 (a negative regulator of insulin signaling) as a key DEG. Overexpression of SOCS3 has been shown to induce insulin resistance and promote fatty acid synthesis [35,36]. These results suggested that CLA might alleviate insulin resistance and disturb glucose homeostasis partly by lowering hepatic D-sedoheptulose 7-phosphate levels and repressing SOCS3 expression. The integration analysis revealed that the hepatoprotective phenotypes conferred by CLA are correlated with molecular alterations in three signaling pathways (MAPK/NF-κB, PPAR, and insulin signaling) and key regulatory nodes (JUN, FASN, and FADS2). Among these, alterations in the MAPK/NF-κB inflammatory cascade are supported by our western blot protein evidence. However, the observed changes in PPAR and insulin signaling are derived from multi-omics profiling. In the absence of targeted functional experiments such as gene interference, receptor inhibition, or cellular pathway perturbation, our data cannot establish that CLA directly modulates or mediates PPAR or insulin-signaling function. Taken together, these correlative multi-omics results describe a hypothetical regulatory network that may partly explain CLA-driven improvements in lipid metabolism disorders, inflammation, and oxidative stress in FLHS chicken livers. It should be emphasized that correlative multi-omics data cannot establish definitive causal relationships. Further functional experiments are required to verify these proposed regulatory cascades.

In the current study, the male Hy-line white chickens were selected as a preliminary FLHS model for rapid mechanistic exploration, but inherent physiological differences limit the extrapolation of conclusions to commercial laying hens. Naturally, FLHS in high-yield layers originates from long-term endogenous estrogen secretion, a high-energy diet, and blocked VLDLy-mediated yolk lipid transport. However, roosters lack ovarian tissue and the vitellogenesis process, and lipid accumulation is merely induced by exogenous estradiol injection without lipid outflow pressure. Although estrogen-activated hepatic lipogenic, inflammatory, and antioxidant pathways are conserved between the two genders, lipid deposition pattern and disease progression are fundamentally different. Therefore, the CLA intervention effect and optimal supplemental dose obtained from this rooster model cannot be directly applied to laying-hen production, and further verification trials on peak-laying hens are required.

## 5. Conclusions

We conducted transcriptome and metabolome analyses to assess the regulatory effect of CLA on hepatic metabolism in chickens with FLHS. Our analysis indicated that CLA reshapes the hepatic metabolic profile, which might be mediated by regulating metabolite production (such as QH2, L-carnitine, linolenelaidic acid, phosphatidylserine, LysoPC, 15s-HPETE, and D-sedoheptulose 7-phosphate), the transcriptional key genes (FASN, JUN, and FADS2), and the activity of core signaling pathways (MAPK/NF-κB, PPAR, insulin signaling). Suppression of the MAPK/NF-κB inflammatory cascade is corroborated by our protein-expression measurements. Nevertheless, the putative engagement of PPAR and insulin signaling remains a correlative observation from multi-omics datasets and requires further functional validation. The above-mentioned correlative molecular network may contribute to the alleviation of lipid and glucose metabolism disturbances, inflammatory responses, and oxidative stress in FLHS chickens. These findings provide novel insights into the pathogenesis of FLHS and identify potential therapeutic targets for the prevention and treatment of fatty liver disease in poultry. This work also delivers correlative molecular evidence supporting CLA as a promising functional feed-additive candidate for FLHS prevention. Nevertheless, further experimental validation is needed to confirm these observations.

## Figures and Tables

**Table 1:** Dietary composition and nutrient concentration levels (air-dry basis) %.

Composition of Diet	Basal Diet	CLA Diet
Soybean meal	17.20	17.20
Corn	71.10	71.10
Premix *	5.00	5.00
CLA	0	0.50
Zeolite powder	6.70	6.20
Total	100.00	100
Nutrient level		
Crude protein, %	14.79	14.79
Metabolic energy, kcal/kg	2751	2788.10
Methionine, %	0.37	0.37
Lysine, %	1.00	1.00
Calcium, %	0.90	0.90
Available phosphorus, %	0.40	0.40

* Supplied with the following nutrients per kg of diet: Cu, 8 mg; Fe, 60 mg; Zn, 50 mg; Mn, 50 mg; I, 0.35 mg; Se, 0.30 mg; NaCl, 3.50 g; VB1, 0.35 mg; VB2, 7.5 mg; niacinamide, 35 mg; VB_5_, 20 mg; VD_3_, 2500 IU; VA, 6000 IU; VK_3_, 5 mg; DL-α-tocopherol acetate, 50 mg; choline, 1 g; biotin, 0.03 mg; folic acid, 0.05 mg; Ca, 9 g; P, 4 g.

**Table 2:** Primers used in real-time quantitative PCR.

Gene Name	GenBank Accession No.	Sequence of Primers (5′-3′)	Product Size (bp)
*β-actin*	NM_205518.1	F: CCAGCCATGTATGTAGCCATCC	182
		R: CACCATCACCAGAGTCCATCAC	
*ADIPOQ*	NM_206991.2	F: GCCAGGTCTACAAGGTGTCA	86
		R: CCATGTGTCCTGGAAATCCT	
*CAT*	NM_001031215.2	F: CCTGACTATGGCGCACGTAT	106
		R: CAGACACACGAGAAGTGGCT	
*DUSP7*	NM_001318409.2	F: CTGCCCATCCGCTCGATTAT	84
		R: GTAGAGCAGTACGGTGTCGG	
*FABP4*	NM_204290.2	F: GACAATGGCACACTGAAGCAG	81
		R: CAGGTTCCCATCCACCACTTT	
*FABP5*	NM_001006346.2	F: GCCATCGACGCGTTTTTAGG	92
		R: CTCATGGCCATACCCACACC	
*FADS2*	NM_001160428.3	F: GGCCTTCCACATCAATCCCA	125
		R: TCCGAAAATCCTCCACCAGC	
*FASN*	NM_205155.2	F: AATCTGCCGTCTGGAACTGAATGG	169
		R: CATCCTGTGACTGGTCGTGTTCTC	
*FOS*	NM_205508.1	F: TGATGTACCAGGGCTTCGCT	94
		R: GACGGGTAGTAGGTGAGGCT	
*HMGCS2*	XM_040677946.2	F: ATCTTCCACACGCCCTTCTG	171
		R: CTTCTCCACCTCCTTGCTGG	
*IL-1β*	NM_204524.2	F: TTCATCTTCTACCGCCTGGACAG	154
		R: GCTTGTAGGTGGCGATGTTGAC	
*IL1R1*	NM_205485.2	F: TCGGAAAGGTCACACTCAGC	75
		R: TCCGTGTTTTCTGGATGGCA	
*IL6*	NM_204628.2	F: CTCGTCCGGAACAACCTCAA	96
		R: GGAGAGCTTCGTCAGGCATT	
*IL8*	NM_205018.2	F: GTAGCTGCTGTCATGGCTCT	98
		R: TCTATGCACTGGCATCGGAG	
*JUN*	NM_001031289.2	F: TCAACTCCGCACCCAACTAC	136
		R: CCCGGCATTTCAGGTACAGT	
*MAP3K8*	XM_040665364.2	F: TCGGATGTGCTCCTGTTTCC	82
		R: CCTTCCCAAAAGCTCCTCGT	
*ME1*	NM_204303.1	F: TCCAGCAGGTGTCACTGAAGATTG	109
		R: GAACGGATGAAGGCTTCGAGGTC	
*NF-κB*	XM_046939918.1	F: GGTGGTATGTGGGAAGGCTTTGG	115
		R: CAGATGCTGGCTTTGTGATGTTGAC	
*Nrf2*	NM_001011678.2	F: CCCAGTCTTCACTGCTCCTC	165
		R: TCAGCCAGCTTGTCATTTTG	
*PPARα*	NM_001001464.1	F: TGCTGTGGAGATCGTCCTGGTC	166
		R: CTGTGACAAGTTGCCGGAGGTC	
*SCD*	NM_204890.2	F: ACCTTAGGGCTCAATGCCAC	89
		R: TCCCGTGGGTTGATGTTCTG	
*SOD1*	NM_205064.2	F: TGACCTCGGCAATGTGACTG	94
		R: GCCAATGATGCAGTGTGGTC	
*TNF-α*	XM_046900549.1	F: CCCAGTTCAGATGAGTTGCCCTTC	94
		R: GCCACCACACGACAGCCAAG	
*TGFB1*	NM_001318456.1	F: GCTCTACATCGACTTCCGCA	126
		R: ACCTTGGTGTACTGCGTGTC	

ADIPOQ, adiponectin; CAT, catalase; DUSP7, dual specificity phosphatase 7; FABP4/5, fatty acid binding protein 4/5; FADS2, fatty acid desaturase 2; FASN, fatty acid synthase; FOS, FBJ osteosarcoma oncogene; HMGCS2, 3-hydroxy-3-methylglutaryl-CoA synthase 2; IL-1β, interleukin-1 beta; IL1R1, interleukin 1 receptor type 1; IL6/8, interleukin 6/8; JUN, Jun proto-oncogene; MAP3K8, mitogen-activated protein kinase kinase kinase 8; ME1, malic enzyme 1; NF-κB, nuclear factor kappa-B; Nrf2, nuclear factor erythroid 2-related factor 2; PPARα, peroxisome proliferator-activated receptor α; SCD, stearoyl-CoA desaturase; SOD1, superoxide dismutase; TNF-α, tumor necrosis factor alpha; TGFB1, transforming growth factor beta 1.

**Table 3:** DEGs in the top 5 enriched pathways.

KEGG Pathway	Upregulated	Downregulated
Metabolic pathways	MGAT5B, CAD, ALPI, INPPL1, LDHD, ALPPL, PRUNE1, GALK1, INPP5J, ADPRM, HSD17B1, AMT, MVD, KHK, FADS1, ELOVL2, RNGL, SMPD1, NDUFA11, ALDH3B1, BCKDHA, NAT8B, HEXDCL, FADS2, FPGS, PIGB, HMGCS2, AGPAT4, NAGLU	GSTZ1, HKDC1, AKR1B1, SCD, AKR1E2, ETNPPL, MAOA, CYP3A4, UGP2, OXCT1, ME1, FASN, ACLY, CYP7A1, LPIN1, PFKFB3, ITPKB, AGXT2, PLOD2, PDE4B, XDH, PSAT1, ACAT2, GSTA3, CDO1, ACKR2, ACMSD, ADH1C, PDE11A, ENSGALG00010002792
Cytokine–cytokine receptor interaction	TGFB1, CCL21, IL13RA1, TNFSF10, IL13RA2, IL17RC, CCR7, MPL, INHBE, ENSGALG00010027340, GDF3	IL1R2, IL1R1, IL1RL1, IL18, CCL4
MAPK signaling pathway	RASGRP3, TGFB1, PTPN7, DUSP7	MAPK11, AREG, EREG, MAP3K8, FOS, DUSP6, IL1R1, EPHA2, HSPA2, FGF23, DUSP8, NR4A1, JUN, DUSP4, DUSP5
PPAR signaling pathway	HMGCS2, FADS2, PPARA	FABP4, FABP5, ME1, SCD, ADIPOQ, ACKR2, CYP7A1
Folate biosynthesis	ALPPL, ALPI, FPGS	AKR1B1, AKR1E2
Biosynthesis of unsaturated fatty acids	ACOT7, FADS1, ELOVL2, FADS2	SCD
Toll-like receptor signaling pathway		MAPK11, SPP1, MAP3K8, NFKBIA, FOS, JUN, CCL4
Fatty acid metabolism	ELOVL2, FADS1, FADS2	ACAT2, SCD, FASN
Insulin signaling pathway	INPPL1	HKDC1, SOCS1, FASN, SOCS3

## Data Availability

The original contributions presented in this study are included in the article. Further inquiries can be directed to the corresponding author.

## Abbreviations

15s-HPETE, 15-Hydroperoxyeicosa-8Z,11Z,13E-trienoate; 15-HETE, 15-hydroxyeicosatetraenoic acid; ADIPOQ, adiponectin; ALT, alanine transaminase; AST, aspartate transaminase; ATF3, activating transcription factor 3; BP, biological process; CAT, catalase; CC, cellular component; CCL21, C-C motif chemokine ligand 21; CLA, conjugated linoleic acids; DAMs, differentially abundant metabolites; DEGs, differentially expressed genes; ELOVL2, elongation of very-long-chain fatty acids 2; FABP, fatty acid binding protein; FADS, fatty acid desaturase; FASN, fatty acid synthase; FOS, Fos proto-oncogene; FLHS, fatty liver hemorrhagic syndrome; GO, gene ontology; GSH-Px, glutathione peroxidase; HDL-C, high-density lipoprotein cholesterol; HMDB, human metabolome database; HMGCS2, 3-hydroxy-3-methylglutaryl-CoA synthase 2; IL13RA2, interleukin 13 receptor subunit alpha 2; INSIG, insulin-induced gene; JUN, Jun proto-oncogene; KEGG, kyoto encyclopedia of genes and genomes; LC-MS, liquid chromatography-mass spectrometry; LDL-C, low-density lipoprotein cholesterol; LIT, linear ion trap; MAPK, mitogen-activated protein kinase; MCODE, molecular complex detection; MDA, malondialdehyde; ME1, malic enzyme 1; MF, molecular function; M-MLV, Moloney murine leukemia virus; NAD, nicotinamide adenine dinucleotide; NAFLD, nonalcoholic fatty liver disease; NFKB, nuclear factor-kappa B; NR4A1, nuclear receptor subfamily 4 group A member 1; OPLS-DA, orthogonal projections to latent structures-discriminant analysis; PCR, polymerase chain reaction; PPAR, peroxisome proliferator-activated receptor; PPI, protein–protein interaction; QH_2_, ubiquinol; QQQ, triple quadrupole; QTRAP, trap mass spectrometer; RIN, RNA integrity number; RNA, ribonucleic acid; SCD, stearoyl-CoA desaturase; SOD, superoxide dismutase; STRING, search tool for the retrieval of interacting genes/proteins; TC, cholesterol; TG, triglyceride; TGFB1, transforming growth factor beta 1; VIP, variable importance in projection.
